# Effects of rumen-native microbial feed supplementation on milk yield, composition, and feed efficiency in lactating dairy cows

**DOI:** 10.1093/jas/skac275

**Published:** 2022-08-30

**Authors:** Ainhoa Valldecabres, Sean P Gilmore, Jordan J Embree, Ivan Z Zhelev, James R Gaffney, Clarisse A Marotz, Fan Yang, Andrew S Izzo, Mallory M Embree, Alfonso Lago

**Affiliations:** DairyExperts Inc., Tulare, CA 93274, USA; Native Microbials Inc., San Diego, CA 92121, USA; Native Microbials Inc., San Diego, CA 92121, USA; Native Microbials Inc., San Diego, CA 92121, USA; Native Microbials Inc., San Diego, CA 92121, USA; Native Microbials Inc., San Diego, CA 92121, USA; Native Microbials Inc., San Diego, CA 92121, USA; Native Microbials Inc., San Diego, CA 92121, USA; Native Microbials Inc., San Diego, CA 92121, USA; DairyExperts Inc., Tulare, CA 93274, USA

**Keywords:** cattle, feed additive, microbial feed supplement

## Abstract

The objective of this study was to evaluate the effects of two rumen-native microbial feed supplements (MFS) on milk production, milk composition, and feed efficiency. A total of 90 multiparous cows between 40 and 60 d in milk were enrolled in a randomized block design study. Within each block (baseline milk yield), cows were randomly assigned to: control (no microbial feed supplementation), MFS1 (0.33 g/kg total mixed ration [TMR] of an MFS containing a minimum of *Clostridium beijerinckii* at 2 × 10^6^ CFU/g and *Pichia kudriavzevii* at 2 × 10^7^ CFU/g), or MFS2 (0.33 g/kg TMR of a MFS containing a minimum of *C. beijerinckii* at 2 × 10^6^ CFU/g, *P. kudriavzevii* at 2 × 10^7^ CFU/g, *Ruminococcus bovis* at 2 × 10^7^ CFU/g, and *Butyrivibrio fibrisolvens* at 2 × 10^7^ CFU/g). Cows were housed in a single group and fed the study diets ad libitum for 270 d. Individual milk yield was recorded using electronic milk meters, and milk fat and protein were measured using optical in-line analyzers at each of two daily milkings. Treatment and treatment by time effects were assessed through multiple linear regression analyses. Treatment effects were observed for milk and energy-corrected milk (ECM) yields, milk fat and protein yields and concentrations, dry matter intake (DMI), and feed efficiency; those effects were conditional to time for milk yield, DMI, and feed efficiency. Overall, milk, ECM, fat, and protein yields were higher for MFS2 compared with control cows (+3.0, 3.7, 0.12, and 0.12 kg/d, respectively). Compared with MFS1, milk yield was higher and protein yield tended to be higher for MFS2 cows (+2.9 and 0.09 kg/d, respectively). In contrast, MFS1 cows produced 0.17 and 0.08 units of percentage per day more fat and protein than MFS2 cows, and 0.07 units of percentage per day more protein than control cows. Dry matter intake and feed efficiency were higher for MFS2 cows compared with MFS1 cows (+1.3 kg/d and 0.06, respectively), and feed efficiency was higher for MFS2 cows compared with control cows (+0.04). Where observed, treatment by time effects suggest that the effects of MFS2 were more evident as time progressed after supplementation was initiated. No effects of microbial supplementation were observed on body weight, body condition score, somatic cell count, or clinical mastitis case incidence. In conclusion, the supplementation of MFS2 effectively improved economically important outcomes such as milk yield, solids, and feed efficiency.

## Teaser

Supplementing dairy cattle with microorganisms native to the rumen improves feed efficiency and the production of milk and milk components.

## Introduction

Improving production efficiency is undoubtedly a priority for the dairy industry with consumers and government policies demanding producers to maximize milk production while minimizing negative environmental impacts. The link between the rumen, the rumen microbiota, and production efficiency is well established. Digestion in the rumen is conducted by the rumen microbial community, degrading and fermenting otherwise nondigestible plant material into molecules useful to the ruminant (Bergman, 1990; Beuvink and Spoelstra, 1992; Dijkstra, 1994). Recent developments suggest that the species composition of the rumen is predictive of dairy cow productivity and the interactions among micro-organisms may play a more significant role than previously considered (Sasson et al., 2017; Xue et al., 2020). Therefore, the ability to alter the rumen microbiome in a precise manner and skew the community towards a state that enables higher feed digestibility and improved animal production is a desirable strategy to improve productivity in the dairy industry (Moraïs and Mizrahi, 2019; Xue et al., 2020). One promising strategy to achieve this is to influence the host’s microbiota by feeding live micro-organisms (Pinloche et al., 2013; Michalak et al., 2021). Most of the microbial feed products on the market today are sourced from environments outside of the rumen; however, recent research has shown that strains native to a given environment are better at colonizing that environment compared with exogenous strains (Russell et al., 2021).

To identify rumen-specific micro-organisms as candidates for a microbial feed supplement (MFS), rumen samples were collected and sequencing libraries were generated for a large and diverse cohort of lactating dairy cows. Sequencing data was then analyzed to identify microbial species associated with positive animal production outcomes (Martino et al., 2018). A diverse range of species had positive associations with performance, leading to the hypothesis that an MFS including multiple micro-organisms will likely influence the rumen more than a single organism. To this end, several strains were isolated from rumen content of a healthy, lactating dairy cow for further study: the cellulolytic micro-organisms *Pichia kudriavzevii* ASCUSDY21 and *Clostridium beijerinckii* ASCUSDY20; and the amylolytic micro-organisms *Butyrivibrio fibrisolvens* ASCUSDY19 and *Ruminococcus bovis* ASCUSDY10. All four species are commonly found in the rumen microbiome of commercially relevant breeds, including Holstein, Jersey, and Nordic Red cows (Wallace, 2019).

*Pichia kudriavzevii*, a budding fungus, has been reported to rapidly degrade cellulose and *other complex polysaccharides* in vitro under simulated rumen conditions (Fernandes et al., 2019). Gomez-Flores et al. (2017) found that *C. beijerinckii* interacts with other complex-carbohydrate degraders synergistically to produce extensive amounts of acetate and butyrate. As a member of the amylolytic group, *R. bovis* is a novel species and one of the few micro-organisms in the rumen capable of degrading resistant starch (Gaffney et al., 2021). While *B. fibrisolvens* is a well-studied rumen microorganism that has gained interest in the field due to its involvement in biohydrogenation (Kepler et al., 1966; Maia et al., 2010) and efficient conversion of linoleic acid to vaccenic acid via CLA isomer pathways not associated with milk fat depression (Baumgard et al., 2000; McKain et al., 2010). Given the synergistic nature of the acetate and butyrate production of *C. beijerinckii* ASCSUDY20 and the rapid degradation of complex polysaccharides by *P. kudriazveii* ASCUSDY21, these microbes were tested in conjunction as a single MFS (MFS1). To assess the impact of additional amylolytic strains on animal productivity, MFS1 was combined with *R. bovis* ASCUSDY10 and *B. fibrisolvens* ASCUSDY19 (MFS2). Here, MFS1 or MFS2 were fed to lactating dairy cows in TMR for 270 d to determine if rumen-native microorganisms can positively influence multiparous cows’ milk yield, milk composition, and/or feed efficiency.

## Materials and Methods

All procedures were approved by the DairyExperts Institutional Animal Care and Use Committee (IACUC Protocol Number: DE20002).

### Research facility

The study was conducted at a research facility from September 2020 to June 2021 (DairyExperts Inc., Tulare, CA). Cows were housed in the same space; a roof-covered loose system pen with compost bedding. Feed mangers were equipped with a feed intake control and measurement system (BioControl, CRFI, Rakkestad, Norway) that allowed for control of access of cows to feed mangers with different diets, access of cows to multiple mangers within the same treatment diet, and measurement of individual cow feed intake, number of visits, and feeding time. The research facility had a TMR preparation area that included feed storage, feed mixer wagon, and concrete floors allowing neat feed handling for the preparation of the different rations. Next to the cows’ housing area there was a double 10 parallel parlor where each stall was equipped with a milk meter (MPC, AfiMilk, Israel) and an optical in-line milk component analyzer (AfiLab, AfiMilk, Israel) that allowed for individual milk yield and composition determination at each milking. The AfiLab system was calibrated once monthly with data on milk composition from the study cows analyzed by Tulare DHIA (Tulare, CA).

### Study cows’ husbandry and adaptation period

A total of 90 Holstein cows between 20 to 40 d in milk (DIM) in their second or third lactation were sourced from a large commercial dairy farm. Upon arrival at the facility, cows were acclimated for a 21-day adaptation period prior to study initiation. Data was collected from the last 3 d of the adaptation period as a baseline covariate.

Cows had ad libitum access to water and TMR daily. The TMR was formulated to meet or exceed the predicted requirements of energy, protein, minerals, and vitamins for cows weighing 659 kg, 2.4 lactations, 28 kg of DMI, 80 DIM, and yielding 41 kg of milk at 3.7% fat and 3.2% protein (NRC, 2001; Table 1). The amount of TMR prepared daily was calculated based on the previous day average intake plus 5%. Cows in the study were milked twice daily at approximately 0500 and 1700 hours.

**Table 1.** Ingredients and nutrient composition of formulated study cows’ diet

Item	% of DM^1^
Ingredient	
Corn silage	25.55
Alfalfa hay	18.36
Rolled corn	11.18
Dried distillers grains	8.78
Cottonseed	7.98
Almond hulls	6.39
Ground corn	4.79
Soybean meal	4.79
Canola meal	4.79
Molasses	1.60
Wheat mill	1.60
Soy plus	0.80
Limestone	0.80
Rumen protected fat	0.64
Palmitic oil	0.64
Sodium bicarbonate	0.48
Sodium sesquicarbonate	0.48
Salt	0.16
Magnesium oxide	0.08
Complexed Zn, Mn, Cu, and Co mix	0.05
Vitamin A, D, and E premix	0.02
Manganese sulfate	0.01
Selenium yeast	0.01
Zinc sulfate	0.005
Copper sulfate	0.002
Ethylenediamine dihydroiodide	0.001
Nutrient composition	
CP	17.65
EE^2^	5.54
ADF	17.97
Ash-free NDF	27.05
NFC	42.13
Starch	21.24
NE_L_ 3X (Mcal/kg)	1.67

Cows were fed a TMR at 60.1% of DM (average of daily determinations during the study period).

Ether extract.

### Experimental design

Using a controlled randomized design, a sample size of 28 cows per treatment group was calculated to allow for the detection of a 3-kg milk difference (SD = 5 kg) among treatments with an alpha level of 0.05 and power of 0.80 using G*Power software (Faul et al., 2007). To account for possible follow-up losses, a total of 30 cows per treatment group were included in the study. Average milk yield from the baseline period was used to sequentially block cows into groups of 3. Within each block, cows were randomly assigned to one of the three treatment groups using a random number generator (Microsoft Excel; Microsoft Corp., Redmond, WA).

Treatments included: 1) control: negative control, no microbial feed supplementation, 2) MFS1: 0.33 g/kg of TMR of GALAXIS (Native Microbials, Inc., San Diego, CA) containing a minimum of *C. beijerinckii* ASCUSDY20 at 2 × 10^6^ CFU/g and *P. kudriavzevii* ASCUSDY21 at 2 × 10^7^ CFU/g, and 3) MFS2: 0.33 g/kg of TMR of GALAXIS FRONTIER (Native Microbials, Inc., San Diego, CA) containing a minimum of *C. beijerinckii* ASCUSDY20 at 2 × 10^6^ CFU/g, *P. kudriavzevii* ASCUSDY21 at 2 × 10^7^ CFU/g, *R. bovis* ASCUSDY10 at 2 × 10^7^ CFU/g, and *B. fibrisolvens* ASCUSDY19 at 2 × 10^7^ CFU/g. All four micro-organisms were sequenced and screened for potential virulence and pathogenicity factors; no regions of concern were identified. The dosage of each MFS micro-organism was determined based on the estimated overall rumen microbial biomass, individual organism growth rate, and fermentation constrains.

A base TMR was prepared once a day for all treatments in a single batch, afterwards, it was divided into three piles in an amount equivalent to the previous day feed intake for each group plus 5%. Then MFS1 or MFS2 was added into the appropriate pile, and the TMR was reloaded into the mixer wagon for mixing and distribution into the respective mangers. To ensure the microbes were homogeneously mixed, three microtracer evaluations were conducted prior to and during the first two months of the study (Supplementary File S1). The viability of the product was measured monthly by anaerobic plating; the average cell counts met the expected minimum dosage at all time points. To avoid cross contamination between batches, after delivering the feed into the mangers for each treatment, approximately 75 kg of Bermuda grass hay were loaded into the mixing wagon and had the augers running for approximately 4 min before discharging it to sweep away the previous batch TMR residues. The potential of cross-contamination was also evaluated using a microtracer and a negligible amount of cross-contamination was observed (Supplementary File S1, Table S1). A total of 48 feed mangers were sequentially assigned in series of three to each of the treatments (16 mangers/treatment): control, MFS1, and MFS2. The TMR with the respective MFS was delivered once a day into the feed mangers, and cows had ad libitum access to any of the 16 mangers containing their assigned treatment. Microbial feed supplementation was initiated at 50 ± 6 DIM and continued to 320 ± 6 DIM.

### Data collection

Individual cow milk yields and composition were measured at each milking and downloaded using AfiFarm software (AfiMilk, Israel). Milk samples for somatic cell count (SCC) determination were collected into vials and taken to Tulare DHIA (Tulare, CA). These samples were collected at a.m. and p.m. milkings 3 d prior to supplementation start, then every Tuesday and Friday at each of the two daily milkings during the study period.

Individual cow feed intake was continuously recorded through the feed intake control and measurement system described above. Representative TMR samples were collected daily for DM determination. Diet ingredients and nutrient composition, estimated by analyzing each individual feed ingredient for its chemical composition and entering the results into the Cornell Net Carbohydrate and Protein System (CNCPS), are presented in Table 1. Daily intake of supplemented microbes was calculated based on daily *as fed* TMR intake, supplemented product inclusion, and minimum concentration of microbes in the product.

A single DairyExperts technician, blinded to cows’ treatment group, scored cows for body condition using a 1 to 5 scale with 0.25 increments (Ferguson et al., 1994) on the last day of the adaptation period, and on days 30, 60, 90, 120, 150, 180, 210, 240, and 270 of the experimental phase. On the same days, cows were also individually weighed after the morning milking using an electronic scale (PS-2000 scale; Salter Brecknell, Fairmont, MN).

Clinical mastitis was defined as abnormal milk from one or more individual quarters during forestripping at any milking. After a clinical mastitis diagnosis, milk from the affected quarter(s) was cultured in order to identify the etiologic agent. Milk cultures were performed at DairyExperts Milk Quality Lab (DairyExperts Inc., Tulare, CA) using aerobic procedures recommended in the Laboratory Handbook on Bovine Mastitis (National Mastitis Council, 2017).

### Statistical analyses

All statistical analyses were performed with SAS (version 9.4; SAS Institute Inc., Cary, NC). Parity (second or third) and DIM at microbial supplementation start were compared among treatments using the chi-squared test and ANOVA with the FREQ and MIXED procedures, respectively. Baseline values were calculated averaging the information from the last 3 d prior to MFS supplementation start.

#### Production outcomes

Outcomes were evaluated as weekly averages generated using the SQL procedure. Weeks 1 to 38 from MFS supplementation start were defined as seven consecutive study days; last week of the experimental phase (week 39) included data from the last five consecutive days of the study. Prior to data analyses, raw data was plotted using the SGPLOT procedure to screen for outliers; no outliers were detected. Daily milk yield was calculated as the sum of both a.m. and p.m. milk weights (kg); ECM was calculated as (0.3246 × kg of milk) + (12.86 × kg of fat) + (7.04 × kg of true protein; NRC, 2001); milk fat and protein concentrations were calculated as the average of both daily milking readings, and their yields were calculated as the sum of both milkings after multiplying each milking milk fat and protein concentrations readings by the respective milking milk yield.

Multiple linear regression was used to analyze production data with the MIXED procedure. All statistical models included the fixed effects of baseline, treatment, time, and treatment by time. Time (week) was included in the models as a categorical variable. For each outcome, the variance-covariance structure leading to the lowest Akaike’s and Bayesian information criterion was used to model the correlation of multiple measures within cow, with cow (subject of the repeated statement) and block as random effects. Unstructured, compound symmetry, autoregressive 1, heterogeneous autoregressive 1, Toeplitz, and Toeplitz heterogeneous were the variance-covariance structures evaluated. The LSMEANS statement with Bonferroni adjustment was used to quantify the association between treatment and the outcome of interest. In order to limit pairwise comparisons to those of interest [among treatments (MFS1 vs. MFS2 vs. Control) and week], customized hypothesis tests were generated using the PLM procedure with the slice option and Bonferroni adjustment. Results are presented as LSM with the corresponding SEM, unless otherwise stated. Overall model fit was assessed with final models’ residuals plots generated with the residual option in the model statement.

#### Dry matter intake and feed efficiency

Daily DMI (kg of feed consumed per cow in an as fed basis times the DM percentage of the TMR fed that day) and feed efficiency (kg of ECM produced per kg of DMI per day per cow) were evaluated as described for production data. Feed intake records > 90 kg/d were considered outliers and removed prior to data analyses (control: *N* = 7; MFS2: *N*= 4). Final model residual plot evaluation revealed seven observations interfering with compliance of the normality and homoscedasticity of residuals assumptions; these observations were excluded from the analyses in order to meet these assumptions (control: *N* = 3; MFS1: *N* = 3; MFS2: *N* = 1).

#### Body weight and body condition score (BCS)

Body weight and BCS data were evaluated as described for production data using study day (30, 60, 90, 120, 150, 180, 210, 240, and 270) as a time categorical variable instead of week.

#### Udder health

Somatic cell count data was log-transformed prior to statistical analysis using the LOG10 function to provide normal distribution of the data, a.m. and p.m. values and values from the same week were averaged; results are reported as Log_10_SCC. Multiple linear regression as described above was used to evaluate the effect of treatment on SCC. The risk of clinical mastitis was evaluated by Log-binomial regression using the GENMOD procedure and the log link function. The LSMEANS statement, with the EXP option when needed, and Bonferroni adjustment were used to quantify the effect of treatment on clinical mastitis and obtain the associated Wald 95% CI. The relative risk represents the ratio of the probability of having a quarter case of clinical mastitis for cows receiving microbial supplementation compared with control cows. Overall model fit was assessed with the goodness-of-fit chi-squared test. The accompanying figures were created with SigmaPlot (version 14.0; Systat Software Inc., San Jose, CA). Statistical significance was declared at *P* ≤ 0.05 and trends at 0.05 < *P* ≤ 0.10.

## Results

Baseline descriptive statistics of cows enrolled in the study are presented in Table 2. No statistically different distributions of parity (*P* = 0.23) and DIM at enrollment (*P* = 0.97) among treatment groups were observed. Two cows were removed from the study at 66 (control: *N* = 1) and 156 DIM (MFS1: *N* = 1) due to hardware disease and injury, respectively. Data collected prior to the removal of the two cows was used in the statistical analyses.

**Table 2.** Descriptive statistics for baseline categorical (proportions) and continuous (mean ± SD) variables from cows at enrollment in the study

Variable	Treatment^1^		
	Control	MFS1	MFS2
Cows, *N*	30	30	30
Parity, %			
Second	67	50	70
Third	33	50	30
DIM, d	50 ± 7	50 ± 6	49 ± 7
Milk yield, kg/d	41.20 ± 5.62	40.72 ± 6.01	41.29 ± 6.11
Energy-corrected milk yield, kg/d	38.23 ± 5.79	37.68 ± 5.64	36.23 ± 6.81
Fat yield, kg/d	1.30 ± 0.22	1.31 ± 0.22	1.21 ± 0.24
Protein yield, kg/d	1.12 ± 0.18	1.07 ± 0.17	1.07 ± 0.22
Fat concentration, %	3.19 ± 0.29	3.34 ± 0.31	3.12 ± 0.36
Protein concentration, %	2.73 ± 0.12	2.72 ± 0.17	2.71 ± 0.15
Log_10_SCC, cells/mL	1.59 ± 0.60	1.61 ± 0.43	1.60 ± 0.55
DMI, kg/d	24.13 ± 4.13	25.59 ± 3.31	23.80 ± 3.13
Feed efficiency, ECM/DMI	1.61 ± 0.27	1.48 ± 0.21	1.55 ± 0.39
BCS	3.06 ± 0.31	3.09 ± 0.36	3.02 ± 0.28
Body weight, kg	655.4 ± 50.4	668.9 ± 61.2	652.4 ± 36.4

Control: no microbial feed supplementation; MFS1: 0.33 g/kg TMR of GALAXIS (Native Microbials Inc.; San Diego, CA) containing a minimum of *Clostridium beijerinckii* ASCUSDY20 at 2 × 10^6^ CFU/g and *Pichia kudriavzevii* ASCUSDY21 at 2 × 10^7^ CFU/g; MFS2: 0.33 g/kg TMR of GALAXIS FRONTIER (Native Microbials Inc.; San Diego, CA) containing a minimum of *C. beijerinckii* ASCUSDY20 at 2 × 10^6^ CFU/g, *P. kudriavzevii* ASCUSDY21 at 2 × 10^7^ CFU/g, *Ruminococcus bovis* ASCUSDY10 at 2 × 10^7^ CFU/g, *and Butyrivibrio fibrisolvens* ASCUSDY19 at 2 × 10^7^ CFU/g.

On average, MFS1 cows’ daily intake of treatment product was 14.59 ± 2.59 g, corresponding to 2.92 × 10^7^*C. beijerinckii* ASCUSDY20 CFU (±5.18 × 10^6^ CFU) and 2.92 × 10^8^*P. kudriavzevii* ASCUSDY21 CFU (±5.18 × 10^7^ CFU; ±SD) per study day. Cows assigned to MFS2 ate on average 14.97 ± 2.63 g of treatment product per day, corresponding to 2.99 × 10^7^*C. beijerinckii* ASCUSDY20 CFU (±5.25 × 10^6^ CFU), 2.99 × 10^8^*P. kudriavzevii* ASCUSDY21 CFU (±5.25 × 10^7^ CFU), 2.99 × 10^8^*R. bovis* ASCUSDY10 CFU (±5.25 × 10^7^ CFU), and 2.99 × 10^8^*B. fibrisolvens* ASCUSDY19 CFU (±5.25 × 10^7^ CFU; ±SD) per study day.

### Production outcomes

Overall treatment effects on production outcomes are summarized in Table 3.

**Table 3.** Least squares means and SEM for production outcomes, DMI, feed efficiency, body weight, and BCS for cows fed two rumen-native microbial feed supplements (MFS1 or MFS2) from 50 ± 6 to 320 ± 6 DIM

Outcome	Treatment^1^			Fixed effects *P*-value^2^		
	Control	MFS1	MFS2	Treatment	Time^3^	Treatment ×Time
Milk yield, kg/d	34.27 ± 0.82^a^	34.33 ± 0.82^a^	37.23 ± 0.82^b^	0.006	<0.001	0.01
ECM yield, kg/d	35.16 ± 0.91^a^	35.76 ± 0.90^a^	38.81 ± 0.90^b^	0.01	<0.001	0.13
Fat yield, kg/d	1.28 ± 0.03^a^	1.30 ± 0.03^ab^	1.40 ± 0.03^b^	0.03	<0.001	0.29
Protein yield, kg/d	1.05 ± 0.03^a^	1.08 ± 0.03^ab^	1.17 ± 0.03^b^	0.01	<0.001	0.26
Fat concentration, %/d	3.86 ± 0.04^ab^	3.95 ± 0.04^a^	3.75 ± 0.04^b^	0.002	<0.001	0.09
Protein concentration, %/d	3.17 ± 0.02^a^	3.24 ± 0.02^b^	3.16 ± 0.02^a^	0.002	<0.001	0.43
Dry matter intake, kg/d	26.11 ± 0.40^a^	26.07 ± 0.40^a^	27.39 ± 0.40^b^	0.02	<0.001	<0.001
Feed efficiency, ECM/DMI	1.38 ± 0.02^a^	1.36 ± 0.02^a^	1.42 ± 0.02^b^	<0.001	<0.001	<0.001
Body weight, kg	712.6 ± 5.5	702.0 ± 5.4	701.3 ± 5.4	0.22	<0.001	0.50
BCS	3.26 ± 0.03	3.28 ± 0.03	3.18 ± 0.03	0.08	<0.001	0.73

Control: no microbial feed supplementation; MFS1: 0.33 g/kg TMR of GALAXIS (Native Microbials Inc.; San Diego, CA) containing a minimum of *Clostridium beijerinckii* ASCUSDY20 at 2 × 10^6^ CFU/g and *Pichia kudriavzevii* ASCUSDY21 at 2 × 10^7^ CFU/g; MFS2: 0.33 g/kg TMR of GALAXIS FRONTIER (Native Microbials Inc.; San Diego, CA) containing a minimum of *C. beijerinckii* ASCUSDY20 at 2 × 10^6^ CFU/g, *P. kudriavzevii* ASCUSDY21 at 2 × 10^7^ CFU/g, *Ruminococcus bovis* ASCUSDY10 at 2 × 10^7^ CFU/g, and *Butyrivibrio fibrisolvens* ASCUSDY19 at 2 × 10^7^ CFU/g.

Models also included the fixed effect of baseline (*P* < 0.001 for all except BCS [*P*= 0.08]) and the random effects of cow and block (baseline milk yield).

Week after microbial supplementation start except for body weight and BCS (study days 30, 60, 90, 120, 150, 180, 210, 240, and 270).

Different superscripts indicate differences at *P* ≤ 0.05 after Bonferroni adjustment.

#### Milk and ECM yield

**Figure 1.**
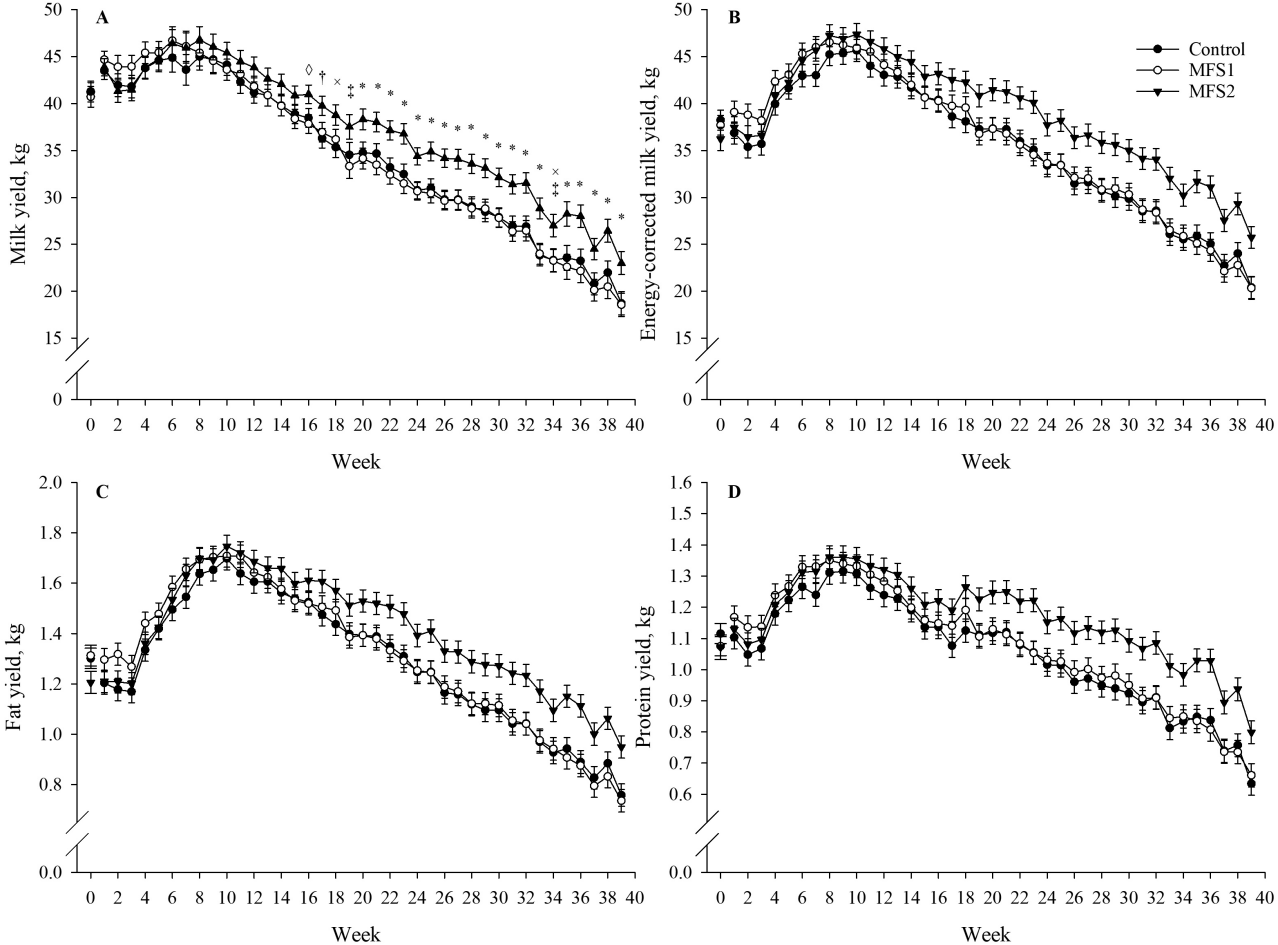
Milk yield (A), ECM yield (B), fat yield (C), and protein yield (D) LSM by treatment and week of study for cows fed two rumen-native microbial supplements (MFS1 or MFS2) from 50 ± 6 to 320 ± 6 DIM. Week 0 represents baseline values. Error bars represent SEM. Symbols indicate: *MFS2 vs. MFS1/control (*P* ≤ 0.05); †MFS2 vs. control (*P* ≤ 0.05); ◊MFS2 vs. MFS1 (*P* ≤ 0.05); ‡MFS2 vs. MFS1 (*P* ≤ 0.10); ×MFS2 vs. control (*P* ≤ 0.10). Fixed effects included in the statistical models represented are: baseline (A, B, C and D: *P* < 0.001), treatment (A: *P* = 0.006; B: *P* = 0.01; C: *P* = 0.03; D: *P* = 0.01), time (week; A, B, C and D: *P* < 0.001), and treatment by time (A: *P* = 0.01; B: *P* = 0.13; C: *P* = 0.29; D: *P* = 0.26). Treatments are: control (no microbial feed supplementation); MFS1 [0.33 g/kg TMR of GALAXIS (Native Microbials Inc.; San Diego, CA) containing a minimum of *Clostridium beijerinckii* ASCUSDY20 at 2 × 10^6^ CFU/g and *Pichia kudriavzevii* ASCUSDY21 at 2 × 10^7^ CFU/g], and MFS2 [0.33 g/kg TMR of GALAXIS FRONTIER (Native Microbials Inc.; San Diego, CA) containing a minimum of *C. beijerinckii* ASCUSDY20 at 2 × 10^6^ CFU/g, *P. kudriavzevii* ASCUSDY21 at 2 × 10^7^ CFU/g, *Ruminococcus bovis* ASCUSDY10 at 2 × 10^7^ CFU/g, and *Butyrivibrio fibrisolvens* ASCUSDY19 at 2 × 10^7^ CFU/g].

Cows assigned to MFS2 produced 3.0 and 3.7 kg/d more milk (*P* = 0.02) and ECM (*P* = 0.01) than control cows, respectively; also, MFS2 cows produced 2.9 and 3.1 kg/d more milk (*P* = 0.02) and ECM (*P* = 0.05) than MFS1 cows, respectively. Milk and ECM yield were not statistically different for control and MFS1 cows during the study period (*P* = 1.00 for both). Overall, treatment effects for milk yield were conditional to time (*P* = 0.01; Table 3). Statistically significant differences between MFS2 and MFS1 and control cows were observed 16 weeks after the initiation of supplementation (Figure 1A). Treatment effects on ECM yield were not statistically conditional to time but a similar effect over time to that for milk yield can be observed in the LSM (*P* = 0.13; Figure 1B). Additional effects included in the models are presented in Table 3.

#### Milk components yield and concentration

Cows assigned to MFS2 produced 0.12 kg/d more of both fat (*P* = 0.04) and protein (*P* = 0.01) than control cows. Also, MFS2 cows tended to produce 0.09 kg/d more protein (*P* = 0.08) than MFS1 cows. However, overall fat yield was not statistically different between MFS2 and MFS1 cows (*P* = 0.14). Additionally, no statistically significant differences were observed for milk fat and protein yield between MFS1 and control cows during the study period (*P* = 1.00 for both). Treatment effects on milk fat and protein yield were not statistically conditional to time (*P* = 0.29 and 0.26, respectively; Figure 1C and D). Additional effects included in the model are presented in Table 3.

**Figure 2.**
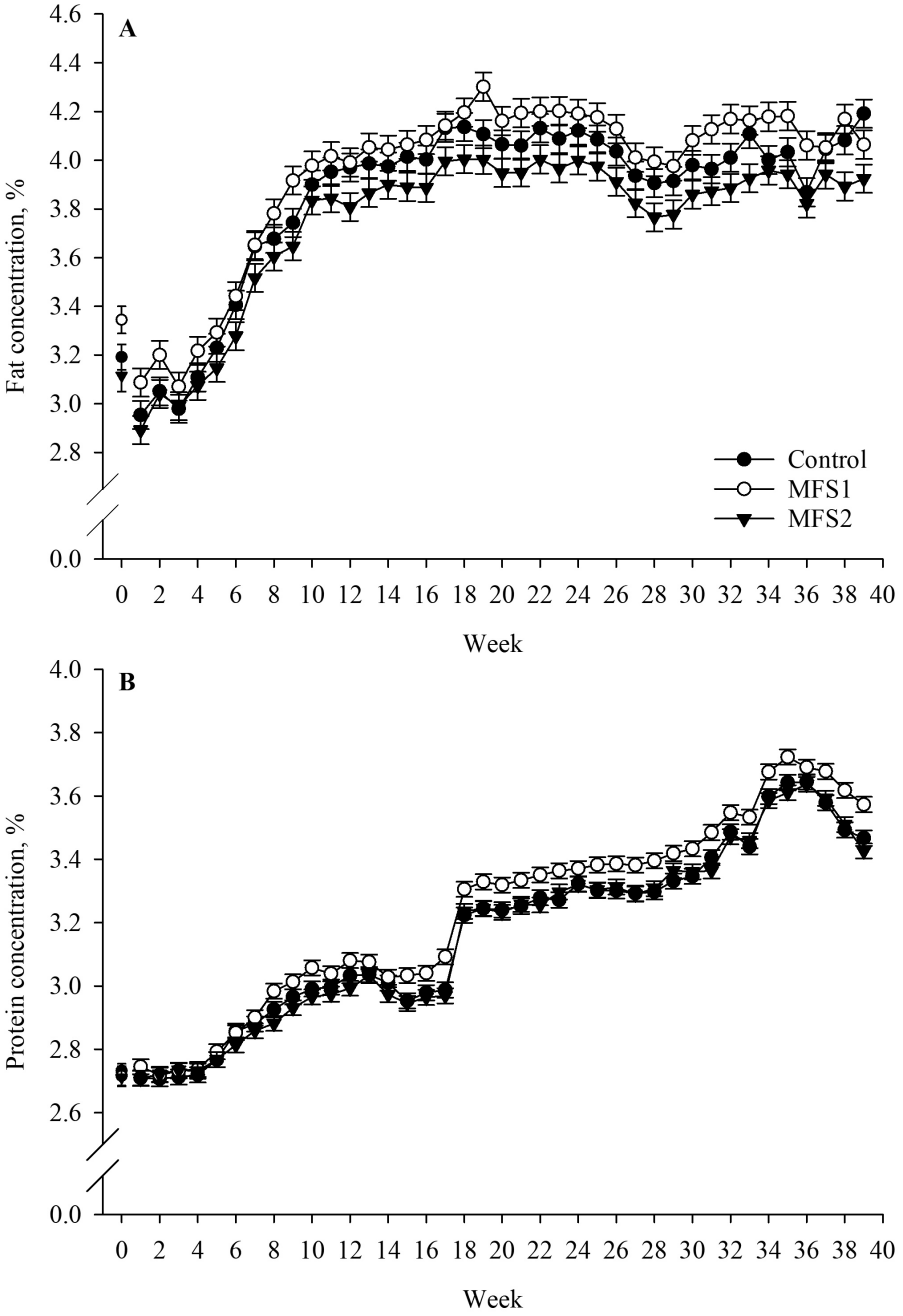
Milk fat (A) and protein concentration (B) LSM by treatment and week of study for cows fed two rumen-native microbial supplements (MFS1 or MFS2) from 50 ± 6 to 320 ± 6 DIM. Week 0 represents baseline values. Error bars represent SEM. Fixed effects included in the statistical models represented are: baseline (A and B: *P* < 0.001), treatment (A and B: *P* = 0.002), time (week; A and B: *P* < 0.001), and treatment by time (A: *P* = 0.09 and B: *P* = 0.43). Treatments are: control (no microbial feed supplementation); MFS1 [0.33 g/kg TMR of GALAXIS (Native Microbials Inc.; San Diego, CA) containing a minimum of *Clostridium beijerinckii* ASCUSDY20 at 2 × 10^6^ CFU/g and *Pichia kudriavzevii* ASCUSDY21 at 2 × 10^7^ CFU/g], and MFS2 [0.33 g/kg TMR of GALAXIS FRONTIER (Native Microbials Inc.; San Diego, CA) containing a minimum of *C. beijerinckii* ASCUSDY20 at 2 × 10^6^ CFU/g, *P. kudriavzevii* ASCUSDY21 at 2 × 10^7^ CFU/g, *Ruminococcus bovis* ASCUSDY10 at 2 × 10^7^ CFU/g, and *Butyrivibrio fibrisolvens* ASCUSDY19 at 2 × 10^7^ CFU/g].

In terms of milk components concentration, cows assigned to MFS1 produced 0.20 and 0.08 units of percentage per day more milk fat (*P* = 0.002) and protein (*P* = 0.004) than MFS2 cows, respectively. Also, MFS1 cows produced 0.07 units of percentage per day more protein than control cows (*P* = 0.01). No statistically significant differences were observed for fat concentration between MFS1 and control (*P* = 0.45) or MFS2 and control cows (*P* = 0.14). Also, no statistically significant differences were observed for protein concentration between control and MFS2 cows during the study period (*P* = 1.00). Treatment effects on fat concentration tended to be conditional to time (*P* = 0.09; Figure 2A), but treatment effects on protein concentration were not statistically conditional to time (*P* = 0.43; Figure 2B). Additional effects included in the model are presented in Table 3.

### Dry matter intake

**Figure 3.**
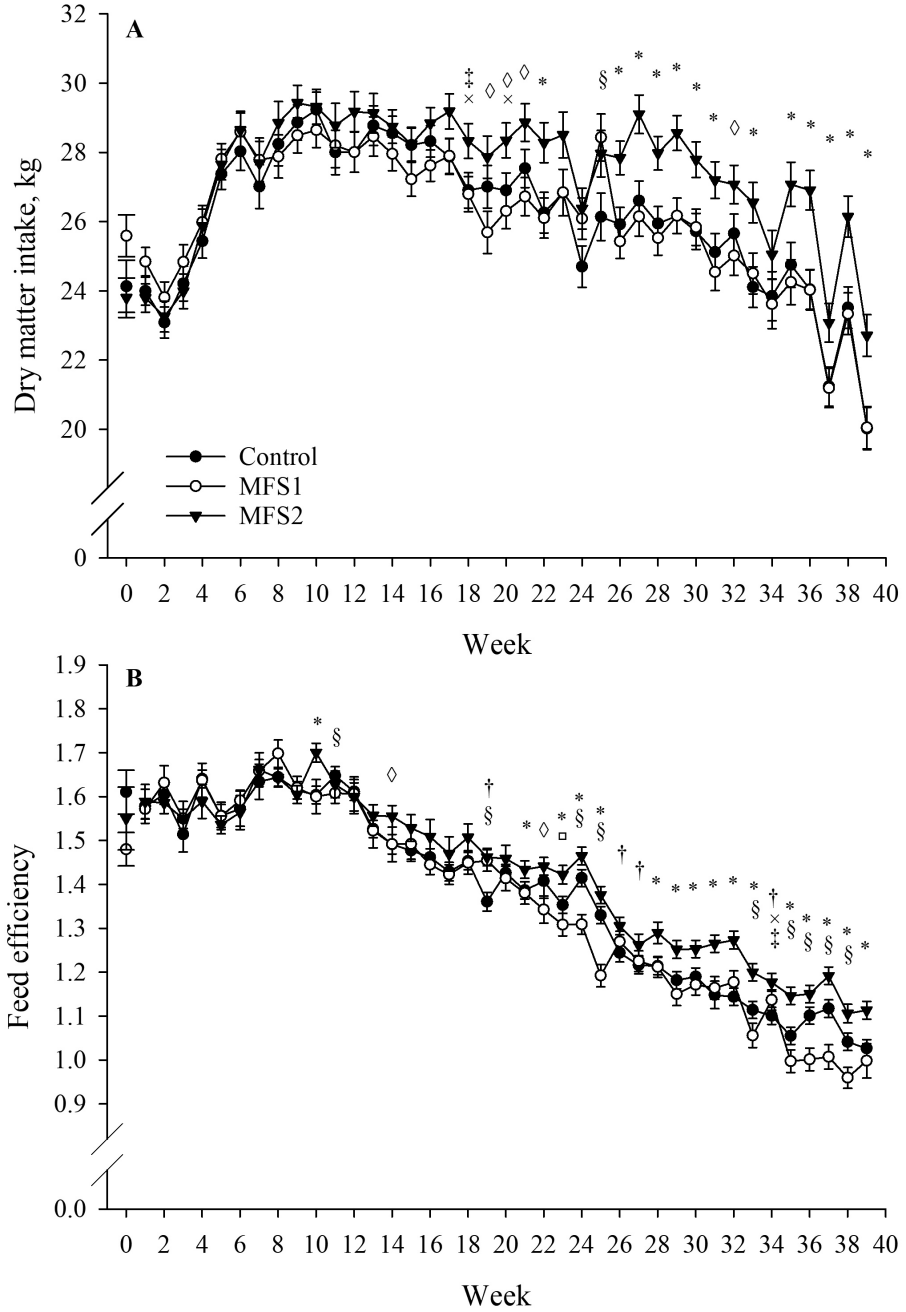
Dry matter intake (A; kg) and feed efficiency (B; ECM/DMI) least squares means by treatment and week of study for cows fed two rumen-native microbial supplements (MFS1 or MFS2) from 50 ± 6 to 320 ± 6 DIM. Week 0 represents baseline values. Error bars represent standard error of the means. Symbols indicate: *MFS2 vs. MFS1/control (*P* ≤ 0.05); †MFS2 vs. control (*P* ≤ 0.05); ◊MFS2 vs. MFS1 (*P* ≤ 0.05); §MFS1 vs. control (*P* ≤ 0.05); ‡MFS2 vs. MFS1 (*P* ≤ 0.10); ×MFS2 vs. control (*P* ≤ 0.10); □MFS1 vs. control (*P* ≤ 0.10). Fixed effects included in the statistical models represented are: baseline (A and B: *P* < 0.001), treatment (A: *P* = 0.02; B: *P* < 0.001), time (week; A and B: *P* < 0.001), and treatment by time (A and B: *P* < 0.001). Treatments are: control (no microbial feed supplementation); MFS1 [0.33 g/kg TMR of GALAXIS (Native Microbials Inc.; San Diego, CA) containing a minimum of *Clostridium beijerinckii* ASCUSDY20 at 2 × 10^6^ CFU/g and *Pichia kudriavzevii* ASCUSDY21 at 2 × 10^7^ CFU/g], and MFS2 [0.33 g/kg TMR of GALAXIS FRONTIER (Native Microbials Inc.; San Diego, CA) containing a minimum of *C. beijerinckii* ASCUSDY20 at 2 × 10^6^ CFU/g, *P. kudriavzevii* ASCUSDY21 at 2 × 10^7^ CFU/g, *Ruminococcus bovis* ASCUSDY10 at 2 × 10^7^ CFU/g, and *Butyrivibrio fibrisolvens* ASCUSDY19 at 2 × 10^7^ CFU/g].

Treatment (*P* = 0.02), time (*P* < 0.001), and treatment by time (*P* < 0.001) effects were observed for DMI (Table 3). Overall, DMI was higher and tended to be higher for MFS2 (27.39 ± 0.40 kg/d) compared with MFS1 (26.07 ± 0.40 kg/d; *P* = 0.04) and control cows (26.11 ± 0.40 kg/d; *P* = 0.04), respectively. Dry matter intake was not statistically different between control and MFS1 cows during the study period (*P* = 1.00). The treatment by time effect on DMI is presented in Figure 3A.

### Feed efficiency

We observed effects of treatment (*P* < 0.001), time (*P* < 0.001), and treatment by time (*P* < 0.001) for feed efficiency (Table 3). Overall, feed efficiency was higher for MFS2 (1.42 ± 0.02) compared with MFS1 (1.36 ± 0.02; *P* < 0.001) and control cows (1.38 ± 0.02; *P* < 0.001). Feed efficiency was not statistically different between control and MFS1 cows during the study period (*P* = 0.26). The treatment by time effect on feed efficiency is depicted in Figure 3B.

### Body weight and BCS

No treatment effects were observed on body weight during the study period (control: 712.6 ± 5.5 kg; MFS1: 702.0 ± 5.4 kg; MFS2: 701.3 ± 5.4 kg; *P* = 0.22); while accounting for the effects of baseline body weight (*P* < 0.001), time (*P* < 0.001), and treatment by time (*P* = 0.50; Table 3). Average BCS tended to be affected by treatment (control: 3.26 ± 0.03; MFS1: 3.28 ± 0.03; MFS2: 3.18 ± 0.03; *P* = 0.08), but treatment group contrasts did not result in statistically significant differences when accounting for the effects of baseline BCS (*P* = 0.08), time (*P* < 0.001), and treatment by time (*P* = 0.73; Table 3).

### Udder health

No statistically significant treatment effect on Log_10_SCC was observed (control: 1.96 ± 0.06 Log_10_SCC/mL; MFS1: 1.92 ± 0.06 Log_10_SCC/mL; MFS2: 1.88 ± 0.06 Log_10_SCC/mL; *P* = 0.47) and no significant treatment by time interaction was observed (*P* = 0.80); while accounting for the effects of baseline Log_10_SCC (*P* < 0.001) and time (*P* < 0.001).

Overall, the incidence of clinical mastitis cases was 18.1% (control: 18.8%; MFS1: 25.0%; MFS2: 10.0%). Risk of clinical mastitis was not statistically associated with treatment (*P* = 0.29). The estimated risk ratios (RR) and associated 95% CI were as follows for the treatment comparisons: MFS1 vs. control (RR = 1.33; 95% CI: 0.52 to 3.41), MFS2 vs. control (RR = 0.53; 95% CI: 0.15 to 1.94), and MFS1 vs. MFS2 (RR = 2.50; 95% CI: 0.73 to 8.55). Organisms identified in clinical mastitis cases are frequently isolated in dairy herds and included: Environmental *Streptococci* (52.9%; control: *N* = 5; MFS1: n = 3; MFS2: *N* = 1), Coagulase Negative *Staphylococci* (17.6%; control: *N* = 2; MFS1: *N* = 1), *Bacillus* spp. (17.6%; control: *N* = 1; MFS1: *N* = 1; MFS2: *N* = 1), *Escherichia coli* (29.4%; MFS1: *N* = 3; MFS2: *N* = 2), and *Pasteurella* spp. (5.9%; control: *N* = 1). No micro-organisms were isolated in one case of clinical mastitis (MFS1: *N* = 1).

## Discussion

The objective of this study was to evaluate the effects of two rumen-native MFS on milk production, milk composition, and feed efficiency in lactating dairy cows. Evaluated MFS1 is comprised of two rumen-native micro-organisms (*P. kudriavzevii* ASCUSDY21 and *C. beijerinckii* ASCUSDY20), while MFS2 is comprised of four rumen-native micro-organisms (*P. kudriavzevii* ASCUSDY21, *C. beijerinckii* ASCUSDY20, *R. bovis* ASCUSDY10, and *B. fibrisolvens* ASCUSDY19). Each individual micro-organism included in the evaluated MFS was isolated from rumen fluid and has metabolic capabilities directly relevant to feed digestibility which may have contributed to the energy availability for lactation and the effects observed on milk production, milk composition, and feed efficiency. More specifically, *P. kudriavzevii* plays a role in metabolizing starch and cellulose and may also be contributing to rumen pH stabilization through lactic acid utilization (Sirisan et al., 2013; Yuangsaard et al., 2013). *C. beijerinckii* ferments an array of plant-derived sugars into acetate, butyrate, and ethanol, and can re-assimilate fermentation gases, including carbon dioxide and *hydrogen, into other valuable metabolites, such as acetate* (Sandoval-Espinola et al., 2017). *R. bovis* facilitates fermentation of starches, including resistant starch, and several sugars into acetate as well as ethanol and *glycerol* (Gaffney et al., 2021). And *B. fibrisolvens* is known to metabolize diverse saccharides derived from feed into VFA, largely butyrate (Hespell et al., 1987; Cotta and Forster, 2006; Emerson and Weimer, 2017). However, further research is necessary to determine the effects of MFS on both rumen and total tract digestibility in vivo.

Higher milk and ECM yields were observed for MFS2 compared with control and MFS1 cows, and milk fat and protein yields were also statistically different between MFS2 and control cows. Higher milk fat and protein concentrations were observed in MFS1 compared with MFS2 cows. A previous study evaluating the effects of supplementing MFS1 reported no statistically significant effects on milk and ECM yields for cows assigned to MFS1 compared with control and suggested that microbial supplementation effects on milk and ECM yields were conditional to cow-level factors such as ECM yield at microbial supplementation start (Goetz et al., 2021). To evaluate the conditional effect described above, the same analysis was conducted in this study, where the treatment by baseline milk or ECM yield interaction was included in its respective model. The interaction was not significant for both milk (*P* = 0.61) and ECM yields models (*P* = 0.54). However, it is important to note that this study included only multiparous (2^nd^ or 3^rd^ parity) which were 50 ± 6 DIM at enrollment (mean ± SD), thus it is plausible that the smaller variability in enrolled cows’ characteristics compared with that in the aforementioned study [primiparous and multiparous, 119 ± 38 DIM at enrollment (mean ± SD)] has prevented us from detecting the interaction.

The observed effects of MFS2 on milk yield were conditional with time, showing statistically significant improvements after 16 wk of supplementation. Numerically, however, weekly means started to diverge after week 6 of supplementation (Figure 1A). Similarly, although not statistically significant, plots of ECM, fat, and protein yields LSM reveal a gap between supplementation start and weekly means divergence (Figure 1B, C, and D). A recent study, also shows effects of MFS on ECM yield and other milk solids content conditional to time (Dickerson et al., 2022). Given the high biomass of the existing rumen microbial community (Matthews et al., 2019), time is likely required for the supplemented strains to integrate with the existing microbial population in the rumen and establish a new microbiome dynamic that influences metabolic function and manifests as a measurable physiological shift in the cow (Weimer et al., 2010; Clemmons et al., 2019). Nevertheless, future colonization studies are required to characterize the accumulation of these strains in the rumen during feeding, and to determine if an increased dose may accelerate production improvements. It is also plausible that the cows’ response to the MFS2 microbes was conditional to DIM, due to physiological states intrinsically associated with lactation stage (Moe, 1981).

Cows assigned to MFS1 had a higher milk protein concentration than MFS2 and control, and a higher fat concentration than MFS2 regardless of time. However, the greater concentrations did not translate to greater overall components yields. This suggests that while the MFS1 micro-organisms contributed to the increase in milk components, they were not sufficient to support a greater overall milk production. Similarly, Goetz et al. (2021) found that milk fat concentration was numerically higher in the MFS1 group, although not statistically different from control. In contrast, Dickerson et al. (2022) did not observe effects of MFS1 or MFS2 supplementation on milk fat and protein concentrations.

The association between milk production and DMI driven by the energy demands to support lactation was evident in our study (NRC, 2001). Over time, DMI trends resembled those of milk yield (Figures 1A and 3A; the observed effects are conditional with time, statistically significant after weeks of supplementation where higher milk yield and DMI are observed for MFS2 cows) and higher DMI was observed for MFS2 compared with MFS1 and control cows. However, despite the higher DMI observed, overall feed efficiency was higher for MFS2 when compared with MFS1 and control cows. Given the observed higher DMI associated with MFS2 in our study, feed efficiency of MFS2 cows was improved as a result of increasing ECM yield. While later stages of lactation are associated with a decrease in feed efficiency (Hurley et al., 2018), the microbes included in MFS2 supported a higher feed efficiency compared with control and MFS1 cows after mid lactation (Figure 3B). In agreement with our findings, Dickerson et al. (2022) reported a trend for a higher feed efficiency on MFS2 compared with MFS1 and control cows. Goetz et al. (2021) reported a trend for higher feed efficiency over the 60 d of microbial supplementation in MFS1 compared with control cows, nevertheless, we did not observe statistically significant differences between MFS1 and control cows in our study. Effect differences between studies may be explained by differences in study design such as MFS dosage, MFS administration strategy, length of supplementation, cows’ parity, or DIM when microbial supplementation was initiated.

Body weight and BCS changes are also factors associated with feed efficiency. When energy from feed intake alone is not sufficient to support maintenance, lactation, or gestation requirements, loss of body weight, reduction in BCS, or both occur (Gross et al., 2011). Despite the increase in ECM, no statistically significant effect of feeding either MFS on body weight or BCS was observed, supporting the idea that the improved feed efficiency is driven by improved digestibility and a better conversion of feed into milk and not due to the use of body reserves. In agreement with our results, Goetz et al. (2021) and Dickerson et al. (2022) did not detect statistically significant effects of MFS on body weight or BCS when compared with control cows. Finally, MFS feeding did not have an effect on milk SCC nor the incidence of clinical mastitis, in agreement with Goetz et al. (2021) and Dickerson et al. (2022).

The observed differences in effects associated with supplementation of the two MFS used in this study are not surprising given their different composition. Supplementation of only two mainly cellulolytic microbes (MFS1) had less of an effect compared with supplementation of four cellulolytic and amylolytic microbes (MFS2). This could be due to the overall smaller dose of rumen-native micro-organisms, to differences in the micro-organisms’ collective metabolic potentials, or potentially both. Further research is required to determine how the dose and composition of a MFS is related to production effects and efficiency in shifting among stable community states in the rumen microbiome.

## Conclusion

Rumen-native microbial supplementation in TMR was associated with higher milk, ECM, fat, and protein yields, and higher feed efficiency when the microbial feed supplement included *Clostridium beijerinckii* ASCUSDY20, *P. kudriavzevii* ASCUSDY21, *Ruminococcus bovis* ASCUSDY10, and *Butyrivibrio fibrisolvens* ASCUSDY19 (four cellulolytic and amylolytic microbes); and with higher milk protein concentration when the microbial feed supplement only included *C. beijerinckii* ASCUSDY20 and *P. kudriavzevii* ASCUSDY21 (two mainly cellulolytic microbes). The absence of statistically significant changes in body weight or BCS during the study period suggests that higher milk yield, ECM yield, and feed efficiency were attained without negatively impacting cows’ body energy reserves. Furthermore, no effects on milk SCC and clinical mastitis incidence were associated with microbial supplementation. Thus, the supplementation of native rumen microbes in TMR is a promising strategy to improve dairy production efficiency.

## Supplementary Material

skac275_suppl_Supplementary_Material

## Abbreviations

BCS: body condition score
DIM: days in milk
DMI: dry matter intake
ECM: energy-corrected milk
MFS: microbial feed supplement
SCC: somatic cell count
TMR: total mixed ration
