# Supplementary material for: Effects of rumen-native microbial feed supplementation on milk yield, composition, and feed efficiency in lactating dairy cows
Source: J Anim Sci. 2022 Aug 30;100(10):skac275. doi: 10.1093/jas/skac275 (PMC9584157; doi:10.1093/jas/skac275)
Supplement: skac275_suppl_Supplementary_Material [file skac275_suppl_supplementary_material.docx]

Supplementary Document 1

**Methods**

***In-feed Product Mixing Evaluation***

To ensure even mixture of the product in the total mixed ration (TMR), we performed a microtracer audit. The microbial feed supplements (MFS) used in this study, MFS1 and MFS2, share the same particle size and inclusion rate. A tracer that is similar in particle sizes, Microtracer^TM^ F-Blue#1 Lake (150-300 microns in diameter) was used in the microtracer audit. According to the manufacturer's instructions, the microtracer was mixed in with the TMR at the same step as the MFS and at the same inclusion rate of 50 grams microtracer per 2,000 lbs of TMR. Three separate sampling events occurred during the study. Upon distribution of TMR to feed bunks, 10 samples each weighing approximately 200 grams were collected from 10 different feed bins along the barn for analysis. TMR samples containing microtracer particles were sent overnight to Micro Tracers, Inc (San Francisco, CA) and the number of particles enumerated by their laboratory. A TMR with a microtracer result of ≥ 80% recovery and <10% coefficient of variation is considered homogeneous mixed.

A fourth sampling event was also conducted to ensure no cross contamination between the control and microbial supplemented TMR batches. Specifically, Microtracer^TM^ F-Blue#1 Lake particles were mixed in with the TMR to simulate a feed preparation for the MFS group. After discharging, approximately 75 kg of Bermuda grass hay were loaded into the mixing wagon and had the augers running for approximately 4 minutes. The Bermuda grass hay was discharged to sweep away the previous batch feed residues. A second batch of TMR was loaded without any microtracer particles to simulate a feed preparation for the Control group. This second batch of TMR was delivered to feed bunks and ten samples (~200 g each) were collected from 10 different feed bins. The samples were sent overnight to Micro Tracers, Inc (San Francisco, CA) for counting.

**Results**

Microtracer audit results are shown in Table S1. All 3 sampling events had good microtracer coverage, with a minimum coverage of 87.79%. The coefficient of variation (CV) of two samples were less than 10% and one event led to a CV of 10.05% (Table S1). The samples from the cross-contamination evaluation showed that the cross contamination is minimum, with 0.32% tracer recovered and a CV of 1.32%. These results suggest that the feed preparation in this study is adequate.

**Table S1.** Microtracer audit results from 3 separate sampling events.

| **Sample Date** | **Tracer Recovery (%)** | **CV (%)** |
| --- | --- | --- |
| 2020-09-10 | 117.98 | 3.42 |
| 2020-09-29 | 87.79 | 10.05 |
| 2020-11-16 | 89.90 | 8.04 |
